# Supplementary material for: The interactome of intact mitochondria by cross-linking mass spectrometry provides evidence for coexisting respiratory supercomplexes
Source: Mol Cell Proteomics. 2017 Dec 8;17(2):216–32. doi: 10.1074/mcp.RA117.000470 (PMC5795388; doi:10.1074/mcp.RA117.000470)
Supplement: Supplemental Data [file supp_RA117.000470_133922_0_supp_23978_fzffwf.zip › spectra_annotation/mito_spectra_annotation/156-1-42-1-7-1.pdf]

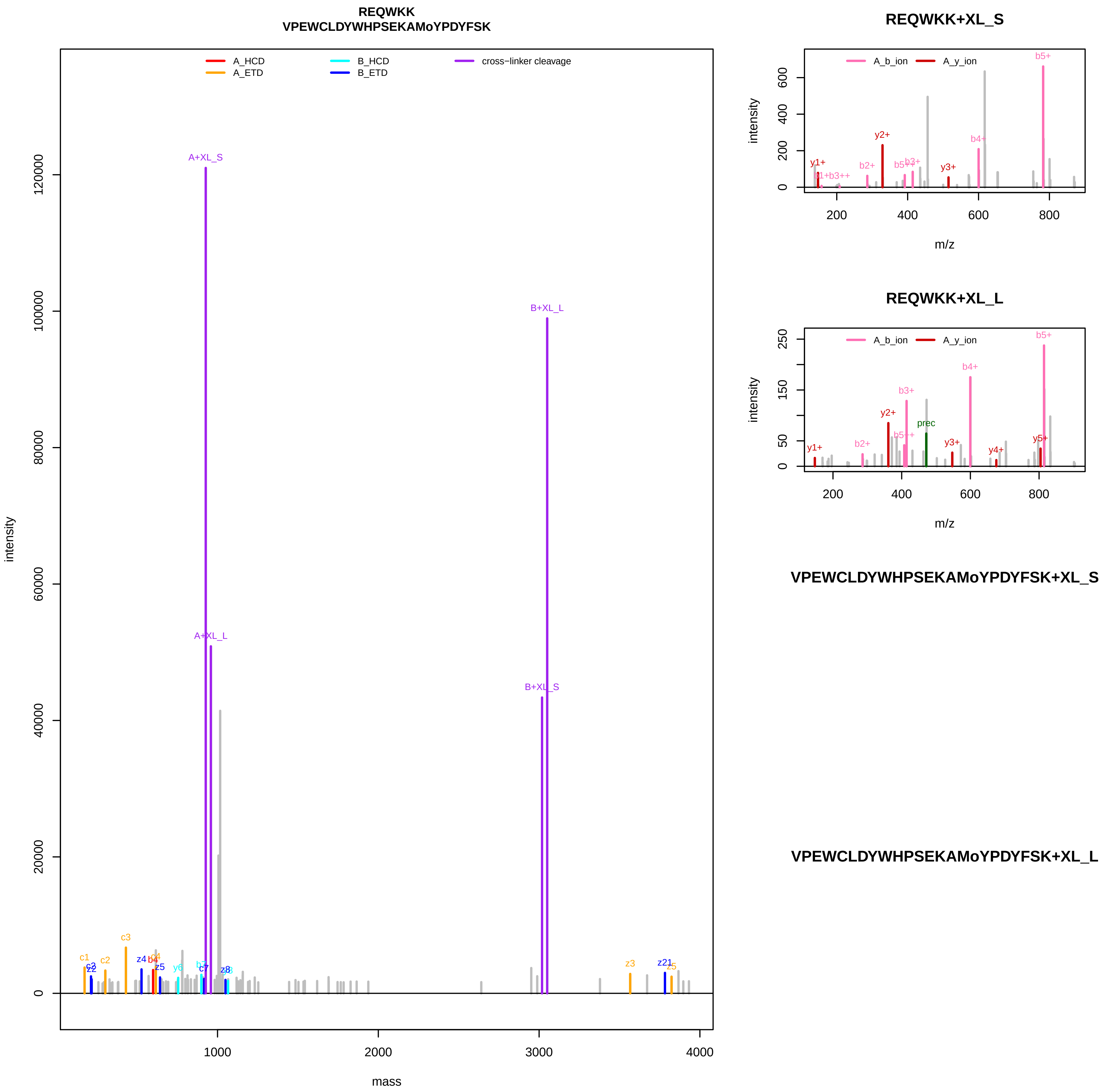
REQWKK
VPEWCLDYWHPSEKAMoYPDYFSK
A_HCD
A_ETD
B_HCD
B_ETD
cross−linker cleavage
A+XL_S
A+XL_L
B+XL_L
B+XL_S
intensity
mass
0
20000
40000
60000
80000
100000
120000
1000
2000
3000
4000
c1
c2
c3
z4
b4
z5
y6
c7
z8
z3
z21
REQWKK+XL_S
A_b_ion
A_y_ion
y1+
y2+
y3+
b2+
b3+
b4+
b5+
b5++
b1+b3++
intensity
m/z
0
200
400
600
800
REQWKK+XL_L
A_b_ion
A_y_ion
y1+
y2+
y3+
y4+
y5+
b2+
b3+
b4+
b5+
b5++
prec
intensity
m/z
0
50
150
250
200
400
600
800
VPEWCLDYWHPSEKAMoYPDYFSK+XL_S
VPEWCLDYWHPSEKAMoYPDYFSK+XL_L
